# Vav1 Sustains the Expression of Insulin, PDX1 and miR-375 During Differentiation of hiPSCs to β Cells: A Potential Target to Improve the *In Vitro* Generation of Insulin-Producing Cells

**DOI:** 10.1007/s13770-025-00777-y

**Published:** 2025-12-11

**Authors:** Marina Pierantoni, Valentina Zamarian, Federica Brugnoli, Silvia Grassilli, Laura Monaco, Marcello Dell’Aira, Valeria Sordi, Valeria Bertagnolo

**Affiliations:** 1 Department of Translational Medicine, University of Ferrara, Via Fossato di Mortara, 70, 44121 Ferrara, Italy; 2 Diabetes Research Institute, IRCCS San Raffaele Hospital, Milan, Italy; 3 Vita-Salute San Raffaele University, Milan, Italy; 4 Department of Environmental Sciences and Prevention and LTTA Centre, University of Ferrara, 44121 Ferrara, Italy

**Keywords:** Vav1, Insulin-producing cells (IPCs), Human induced pluripotent stem cells (hiPSCs), Type 1 diabetes (T1D)

## Abstract

**Backround:**

Human-induced pluripotent stem cells (hiPSCs) have emerged as a promising source of transplantable insulinproducing cells (IPCs) to restore insulin levels in Type 1 Diabetes (T1D) patients. Despite progress, obtaining fully functional β cells from hiPSCs remains challenging, underscoring the need to better understand the intracellular mechanisms involved. We investigated here the potential role of Vav1, a multidomain protein that we identified as crucial for the maturation of human biliary stem cells (hBTSCs) into β-like cells and in the trans-differentiation of pancreatic adenocarcinoma (PDAC) cells into IPCs;

**Methods:**

Levels and subcellular localization of Vav1 were investigated throughout a seven-step differentiation process of hiPSCs to β cells. Vav1expression was forcedly modulated in pancreatic progenitors, and the potential effects were evaluated on insulin production and on PDX1, miR-375, and Akt, key regulators of β cells generation; RESULTS. Vav1 showed dynamic modulation, with pancreatic precursor cells requiring adequate levels of the protein to generate IPCs.

**Results:**

Vav1 sustains the expression of PDX1, a primary regulator of insulin expression, and of its target miR-375, essential for determining β cell mass. Furthermore, Vav1 reduction correlated with increased activation of Akt, which regulates cell survival and insulin secretion in β cells and is down-regulated by miR- 375.

**Conclusion:**

Our findings suggest the existence of a Vav1/PDX1/miR-375/Akt axis as part of the complex network orchestrating the generation of functional β cells. These insights indicate that strategies aimed at specifically modulating Vav1 levels may positively impact the generation of IPCs in vitro and, ultimately, β cell replacement therapy for T1D.

## Introduction

Type 1 diabetes (T1D) is an autoimmune disease characterized by the destruction of insulin-producing cells in the pancreas, necessitating the intake of external insulin to manage the condition and ensure survival [1]. In 2021, approximately 8.4 million people worldwide had T1D, with 64% (5.4 million) aged between 20 and 59 years. Around 0.5 million new cases were diagnosed, and many undiagnosed individuals faced higher mortality. Predictions for 2040 indicate an increase in T1D cases, particularly in low and lower-middle-income countries [2].

Given the rising number of individuals affected by T1D and the associated management costs, developing more efficient therapies is essential to address this growing global health challenge. Furthermore, T1D is associated with acute and chronic complications in both young people and adults, such as diabetic ketoacidosis (DKA), diabetic retinopathy, diabetic nephropathy, cardiovascular disease and hypertension [3, 4] that can complicate the effective management of the pathology.

Currently, T1D is treated with exogenous insulin administration; however, this approach is far from providing physiological metabolic control and is often characterized by frequent episodes of hyperglycemia and hypoglycemia [3]. A more recent approach focuses on pancreatic islet transplantation, which aims to restore endogenous insulin production and has a 90% success rate one-year post-transplant. Despite its promise, this method is limited by the scarcity of donors and the need for long-term immunosuppression [5, 6]. To address these issues, various protocols have been developed to generate insulin-producing cells via complex, multi-step differentiation processes that mimic the natural maturation of pancreatic β cells. Among these, pluripotent stem cells, such as embryonic stem cells (ESC), or induced pluripotent stem cells (iPSC) can be induced to differentiate *in vitro* into insulin-producing cells with established protocols that mimic endocrine pancreas development [7–11].

In the context of developing insulin-producing cells, the use of human iPSCs (hiPSCs) offers significant advantages over ESCs, representing a promising alternative in regenerative medicine and diabetes research [12–14]. Nevertheless, despite recent improvements, the differentiation of the hiPSCs into functional β cells *in vitro* is not homogeneous, and cells that retain stem properties can pose a problem after transplantation [15]. This indicates that a better understanding of the intracellular mechanisms involved in the maturation process is required to obtain fully functional β cells from pluripotent precursors.

Among the signaling molecules involved in generating insulin-producing cells, the multidomain protein Vav1 recently emerged as an interesting element. Despite the well-established role of Vav1 in hematopoietic cells [16–18], its expression in solid tissues has mainly been studied in tumors, including exocrine pancreas tumors [19–22]. In both leukemia and breast tumor-derived cells, Vav1 is involved in modulating gene transcription, cooperating in the regulation of miRNAs and proteins levels [23–26]. In both breast cancer and pancreatic ductal adenocarcinoma (PDAC) cells, Vav1 downregulated the expression and/or activation of specific Akt isozymes [27–29], crucial molecules in sustaining tumor malignancy [30], but also involved in insulin production and secretion through a complex network of signaling pathways [31]. In mature β cells, Akt regulates glucose metabolism, cell survival, proliferation, apoptosis and insulin secretion through the mTORC2/Akt pathway by targeting Mapkap1 expression through miR-375 [32], known for its crucial role in modulating β cell mass and controlling insulin production and secretion during pancreatic differentiation both *in vitro* and *in vivo* [33, 34].

In recent years, we revealed Vav1 expression in some insulin-producing cells of the human adult pancreas and demonstrated that adequate levels of the protein are crucial for the early stages of maturation of human biliary tree stem/progenitor cells (hBTSCs), as well as in the trans-differentiation process of PDAC derived cells, into insulin-producing cells [35]. In the latter cell model, we demonstrated that adequate levels of Vav1 are necessary for the expression of Pancreatic Duodenal Homeobox-1 (PDX1) [35], a transcription factor playing a pivotal role in β cell development as a primary regulator of insulin gene expression [36, 37].

Based on these findings, the main goal of our study was to assess whether Vav1 participates in specific stages of hiPSCs differentiation into insulin-producing cells, focusing on its potential role in modulating molecules with key roles in β cell development and maturation. We revealed the existence of a Vav1/PDX1/miR-375/Akt axis that may regulate insulin biosynthesis and could represent a target for strategies aimed at improving the efficiency of *in vitro* β cell generation.

## Material and methods

### Differentiation of iPSCs into insulin-producing cells

Human iPSC clones were generated by reprogramming CD34-enriched blood cells from healthy subjects using the Sendai virus technology (CytoTune-iPS Sendai Reprogramming Kit, Thermo Fisher Scientific, Waltham, MA, USA) [38, 39]. Cells were maintained at 37 °C, 5% CO_2_ in Essential 8 Basal Medium (Gibco, Waltham, MA, USA) supplemented with 1% of Penicillin/Streptomycin (Lonza, Basel, Switzerland). Cells were passed using EDTA 0.5 mM (UltraPure, ThermoScientific) once 80% confluence was reached. Differentiation to insulin-producing cells started when cell confluency reached 50–60% and was obtained through a 7-step differentiation process lasting a total of 25 days, following an already established protocol [40]. Cells were maintained in a controlled environment at 37 °C with 5% CO_2_, and regular screenings ensured the absence of contamination.

Markers specific to each differentiation stages were evaluated, including OCT4, CXCR4, NKX6.1, PDX1, INS and GCG by flow cytometry, as previously reported [40]. Cells were detached with 1× Trypsin (Lonza) or Accutase (SCR005, Merck Millipore, Milan, Italy) and stained at different stages during the differentiation with stage specific markers. For extracellular staining, cells were washed with FACS buffer (PBS + 0.2% BSA), labelled with the antibodies and fixed with Cytofix/Cytoperm (BD Biosciences, Franklin Lakes, NJ, USA). For the intracellular staining, cells were fixed with Cytofix/Cytoperm, washed with FACS buffer and then permeabilized with BD Phosflow Perm Buffer III (BD Biosciences). After permeabilization, cells were marked with the specific antibodies and examined on a FACS Canto cytometer (BD Biosciences). Results were analyzed with FlowJo 10.8 Software (BD Biosciences).

To validate the physiological competence of cells at the final stage of the differentiation process (iβ), their ability to secrete insulin in response to glucose stimulation was assessed through dynamic perifusion, as previously reported [39, 40]. Briefly, dynamic stimulation of iβ cells was performed on an automated perifusion system (BioRep Perifusion V2.0.0, USA). For each line, 100 clusters were picked and stimulated with HEPES-buffered solution (125 mmol/l NaCl, 5.9 mmol/l KCl, 2.56 mmol/l CaCl2, 1 mmol/l MgCl2, 25 mmol/l HEPES, 0.1% wt/vol BSA, pH 7.4) supplemented as follows: 0.5 mmol/l glucose; 11 mmol/l glucose plus 30 mmol/l KCl. Insulin content was quantified by ELISA kit (Mercodia, Sweden).

### Modulation of Vav1 expression

To assess the optimal conditions for modulating Vav1 expression in hiPSCs induced to differentiate into insulin-producing cells, various liposome mixtures, as siPORT™ NeoFX™ Transfection Agent, Lipofectamine™ RNAiMAX and Lipofectamine™ 2000 (Thermo Fisher Scientific), and different transfection times (24 h, 48 h, 72 h) were tested. To evaluate transfection efficiency, a non-silencing fluorescein-labeled Control siRNA or a pEGFP plasmid (Cell Signalling Technology Danvers, MA, USA) were used. The incubation with 1 mg/ml Lipofectamine™ 2000 for 48 h resulted the condition ensuring the higher transfection efficiency, reaching at least 35% of cells.

To reduce the expression of Vav1, 24 h after seeding, 8 × 10^5^ cells at the “pancreatic progenitors” (PP, 16 days) stage were incubated in 500 μl of Opti-MEM™ with 150 pMol of specific siRNAs (Vav1 siRNAs, Santa Cruz Biotechnology, Heidelberg, Germany). After 4 h, the inoculum was removed, and the culture medium was replaced with the one specific for the achieved differentiation stage [40]. The transfected cells were incubated at 37 °C in a 5% CO2 atmosphere for 48 h and then subjected to immunochemical, immunocytochemical, and RT-qPCR analysis.

To induce the over-expression of Vav1, 24 h after seeding, cells at the “endocrine progenitors” (EP, 19 days) stage were transiently transfected with 2.0 μg of a pEF plasmid expressing the entire sequence for human Vav1 (pEF Vav-myc plasmid, kindly provided by Dr. Germani, ICGM-Hopital Cochin-Inserm, Paris, France), following the above reported protocol optimized for silencing.

Non-silencing control siRNAs (Santa Cruz Biotechnology) and an empty vector (Thermo Fisher Scientific) were used as controls.

### Immunochemical analysis

Total lysates from cells under the different experimental conditions were separated on 8.5% polyacrylamide denaturing gels and blotted onto nitrocellulose membranes (GE Healthcare Life Science, Little Chalfont, United Kingdom), which were reacted with antibodies directed against Vav1 (16364-1-AP Proteintech, Manchester, UK), PDX1 (AF2419 R&D Systems, Minneapolis, MN, USA), pSer474-Akt2 (8599S) and Akt2 (3063 s) from Cell Signaling Technology, pSer473-Akt1 (05-736 Merck Millipore, Milan, Italy), Akt1 (sc‐377457) and GAPDH (sc-47724) from Santa Cruz Biotechnology, following previously reported procedures [25, 35]. Membranes were then incubated with peroxidase-conjugated secondary antibodies, and the immunocomplexes were detected by using a WESTARNOVA2.0 kit (Cyanagen, Bologna, Italy). The chemiluminescence derived bands were captured with Image Quant TM LAS4000 biomolecular imager (GE Healthcare Life Science) and quantified with Image Quant TL software v7.0 (GE Healthcare Life Science), as previously reported [41].

### Immunocytochemical and confocal analysis

The cells at the PP, EP and iβ stages, growing as aggregates characteristic of advanced differentiation stages, were dissociated using Accutase (SCR005, Merck Millipore), fixed with freshly prepared 4% paraformaldehyde, and reacted with a specific primary antibody against Vav1 (16364-1-AP Proteintech) or/and Insulin (C12 sc-377071 Santa Cruz Biotechnology), and against PDX1 (AF2419 R&D Systems), for 3 h at room temperature in Net Gel solution (150 mM NaCl, 5 mM EDTA, 50 mM Tris–HCl pH 7.4, 0.05% NP40, 0.25% Carrageenan Lambda gelatine, and 0.02% Na azide), and then labelled with FITC and/or TRITC-conjugated secondary antibodies (Thermo Fisher Scientific) in the dark at room temperature. After washes with NETgel and PBS, all samples were incubated with 0.5 µg/mL 4′,6-diamidino-2-phenylindole (DAPI), dried with ethanol, and mounted in glycerol containing 1,4-diazabicyclo [2.2.2] octane (DABCO) to delay fading, following previously described procedures [35].

Fluorescence staining was analyzed with a Nikon Ci-L fluorescence microscope (Nikon, Melville, NY, USA). Images were acquired with a DS-Qi2Mc digital camera connected to the NIS-Elements D software (Nikon). Regions containing approximately 50–100 cells were selected, and the fluorescence intensity per cell was quantified (NIS-Elements BR Imaging Software), excluding non-specific signals.

For confocal analysis of Vav1 intracellular distribution, stained cells were observed with an Olympus FV3000 confocal microscope (Olympus Corporation, Tokyo, Japan) equipped with a 63× oil immersion objective (N.A. 1.4) by taking z-series of 0.42 μm each to capture the entire volume of the cells. To measure Vav1 nuclear staining, digitized images were analyzed with ImageJ software (http://rsb.info.nih.gov/ij/), and fluorescence values were expressed as the mean of integrated density (IntDen) of at least 20 nuclei in three different areas.

### Real-time qPCR

High-quality RNA, including small RNAs, was extracted from cells using miRNeasy Micro Kit (Qiagen SpA Italia, Milan, I), according to the manufacturer's instructions.

To measure Vav1 mRNA, RT-qPCR was performed using the qPCRBIO SyGreen Mix Lo-ROX (Resnova s.r.l., Roma, Italy) on a QuantStudio™ 3 Real-Time PCR System (Thermo Fisher Scientific). The following primers for Vav1 were used: 5′-ACGTCGAGGTCAAGCACATT-3′ forward and 5′-GGCCTGCTGATGGTTCTCTT-3′ reverse. Levels of Vav1 were normalized to RPL32 content (5′-CATCTCCTTCTCGGCATCA-3′ forward and 5′-AACCCTGTTGTCAATGCCTC-3′ reverse), as previously reported [26].

MiR-375 expression was evaluated by RT-qPCR using TaqMan Assays (ID 000564, Thermo Fisher Scientific) as previously described [25]. MiR-375 expression levels were normalized to U6 snRNA (ID 001973, Thermo Fisher Scientific). All reactions were performed in triplicate, and the experiments were repeated 3 times.

### Statistical analysis

The results are presented as mean ± standard deviation (SD), calculated from three independent experiments. Statistical analysis was performed using the Student’s T-test for comparisons between two groups, employing the GraphPad Prism software version 6.0. P-values less than 0.05 were considered statistically significant.

## Results

### Expression of Vav1 during differentiation of hiPSCs to iβ

**Fig. 1:**
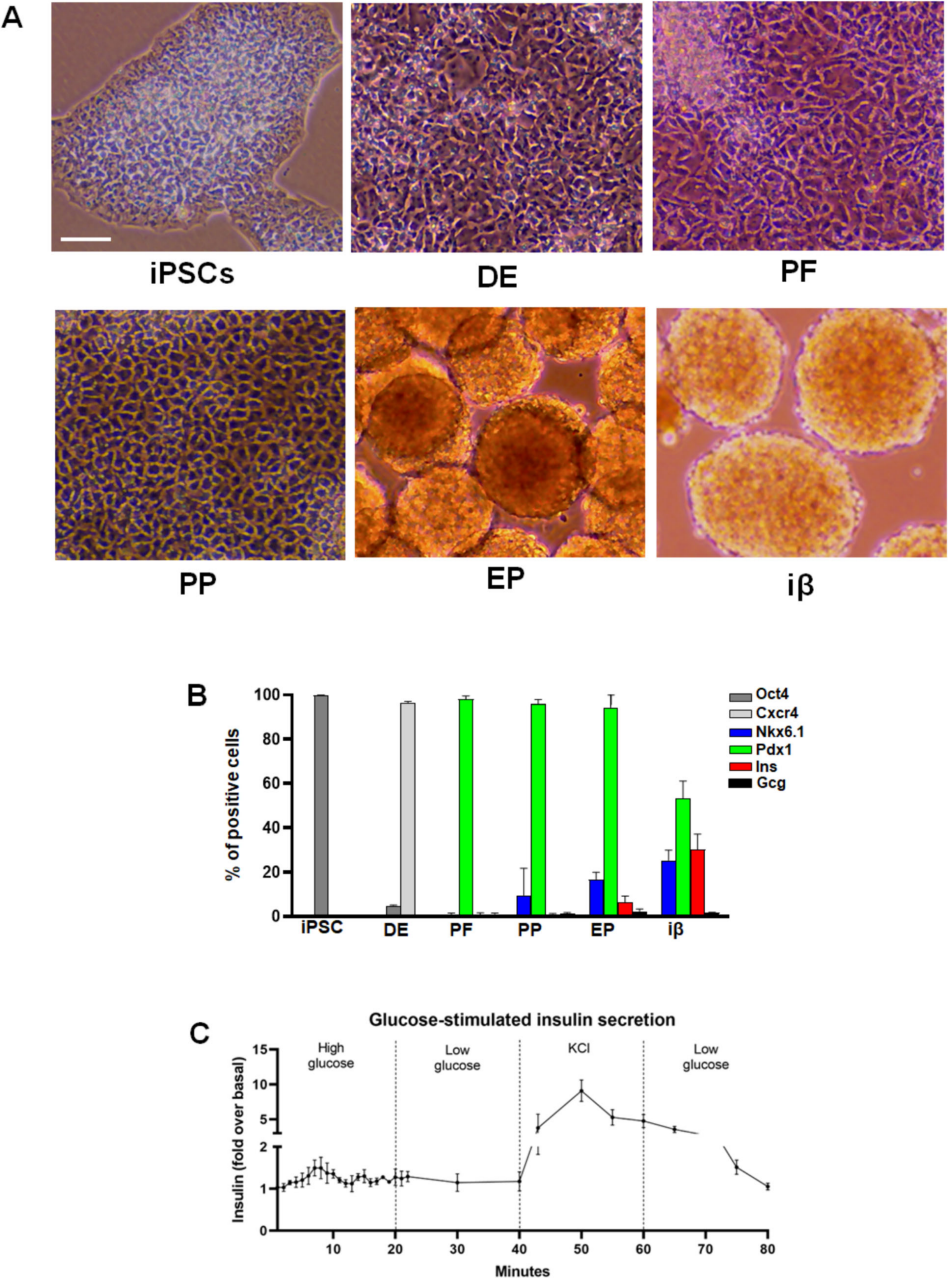
Differentiation of induced pluripotent stem cells (iPSC) into β cells. **A** Representative phase-contrast microscopy images showing cells morphology at various stages of iPSC differentiation into β cells. DE: definitive endoderm, PF: posterior foregut, PP: pancreatic progenitors, EP: endocrine progenitors, iβ: insulin-producing cells. Scale bar: 50 µm. **B** Analysis of key markers used to characterize the various stages of β cell differentiation from iPSCs, as determined by flow cytometry. The percentages of positive cells are shown. (**C**) Glucose-stimulated insulin secretion (GSIS) to assess iβ cell functionality. Cells were stimulated with 0.5 mM Glucose (Low glucose), 11 mM Glucose (High glucose) and 3 mM KCl. Insulin is expressed as fold over basal. All the data represents the mean of three independent experiments ± SD

The differentiation of human iPSCs into pancreatic β cells was achieved using a slightly modified version of a previously described procedure [40] that drives cells through definitive endoderm, primitive gut tube, posterior foregut, pancreatic progenitor, and endocrine progenitor stages, culminating in the generation of insulin-producing cells (iβ) after 25 days. As expected [40], cells transitioned from adherent to suspended (Fig. 1A) and expressed surface markers specific to each maturation stage (Fig. 1B). Notably, the final β-like stage is characterized by cell aggregates resembling pancreatic islets (Fig. 1A), with at least 30% of cells being insulin-positive and glucagon-negative (Fig. 1B), confirming the efficiency of the differentiation procedure. Moreover, glucose-stimulated insulin secretion (GSIS) assays confirmed the physiological competence of the iβ cells (Fig. 1C).

**Fig. 2:**
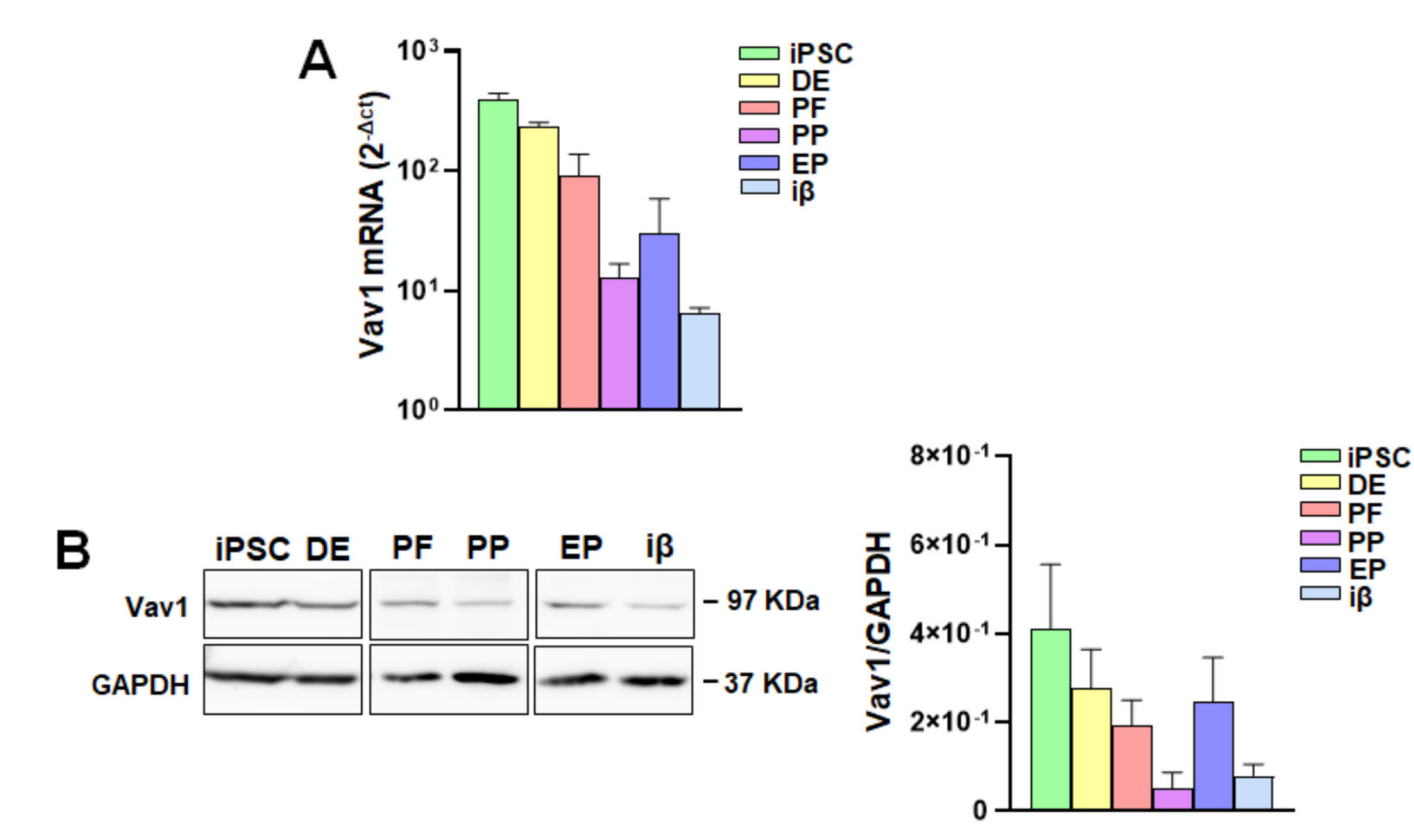
Vav1 expression at distinct stages of iPSC differentiation into β cells. **A** RT-qPCR analysis of Vav1 mRNA. Values were obtained using the 2^−ΔCt^ method. **B** Representative Western blot analysis of total cell lysates at various differentiation stages using a specific anti-Vav1 antibody; protein quantification based on densitometric analysis of bands normalized to GAPDH is shown on the right. Data represents the mean of three independent experiments ± SD

To establish a potential role for Vav1 in the differentiation process of iPSCs into insulin-producing cells, both Vav1 mRNA and protein levels were measured at various stages of the maturation process. RT-qPCR analysis revealed important levels of Vav1 mRNA in precursor cells, which progressively decreased during differentiation, reaching a minimum at the pancreatic progenitor (PP) stage. Subsequently, an increase in Vav1 expression was observed in cells at the endocrine progenitor (EP) stage, followed by a further reduction during the last phase (iβ) of the differentiation (Fig. 2A).

Dynamic changes in Vav1 expression throughout the differentiation of iPSCs into insulin-producing cells were confirmed by Western blot analysis of total cell lysates, proving the trend observed at the mRNA level and indicating that a peak of Vav1 protein characterizes the transition of pancreatic precursors towards the endocrine lineage (Fig. 2B).

**Fig. 3:**
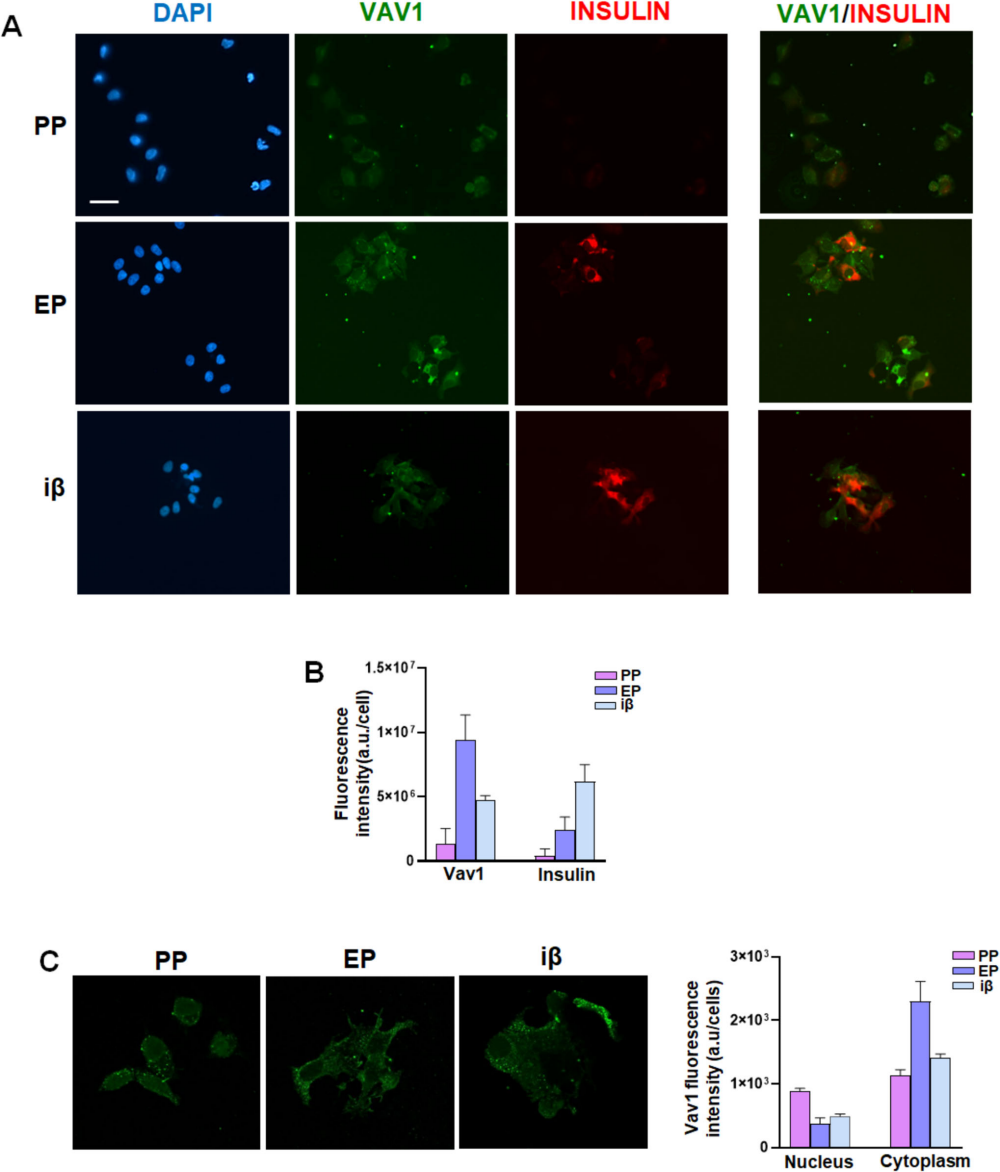
Immunocytochemical analysis of Vav1 and insulin in PP, EP, and iβ cells. **A** Representative images of simultaneous immunocytochemical analysis with antibodies against Vav1 and insulin in dissociated PP, EP, and iβ cells, counterstained with DAPI to highlight the cell nucleus. On the right, the overlay of Vav1 (green) and insulin (red) is shown, with co-localization resulting in orange/yellow. Scale bar: 50 μm. **B** Fluorescence intensity of Vav1 and insulin in the same cell populations, as determined by images analysis. **C** Representative fluorescence images of Vav1 acquired by confocal microscopy. Intensity of nuclear and cytoplasmic Vav1 is shown on the right. Data represents the mean of three independent experiments ± SD

Based on findings obtained by RT-qPCR and Western blot analyses, we focused our investigation on pancreatic precursor cells adopting the endocrine fate. We explored the intracellular localization of Vav1 and its potential correlation with insulin levels using immunocytochemical analysis. As shown in Fig. 3A, B, despite heterogeneous populations, Vav1 fluorescence intensity confirmed the increased protein amount in cells at the EP stage, apparently mainly in their cytoplasm, and the decrease of Vav1 was confirmed in cells at the iβ stages (Fig. 3A). The intracellular localization of Vav1 was further examined by confocal analysis of fluorescent cells, allowing us to distinguish between cytoplasmic and nuclear Vav1. As reported in Fig. 3C, confocal analysis confirmed the presence of Vav1 in the nuclear compartment, with higher levels in PP cells compared to EP and iβ stages, which showed similar amount. Conversely, cytoplasmic Vav1 levels were predominant and reflected the overall protein peak in EP cells (Fig. 3C).

Regarding insulin, fluorescence staining confirmed the progressive increase in the number of positive cells from PP to iβ (Fig. 3A), as revealed by cytofluorometric analysis (Fig. 1B). In both EP and iβ stages, positive cells exhibited varying levels of insulin, apparently not correlated with the amount of Vav1, likely indicative of different maturation levels reached by differentiating β-like cells (Fig. 3A).

### Effects of Vav1 modulation on insulin levels

To investigate the potential effects of Vav1 on the generation of insulin-producing cells from iPSC, we modulated its expression to prevent the increase during the transition from PP to EP and to counteract its decrease at the end of the differentiation process (from EP to iβ). Vav1 silencing was performed in cells at day 16 of the differentiation process, before the peak, while overexpression was induced in cells at day 19, before the Vav1 decrease.

**Fig. 4:**
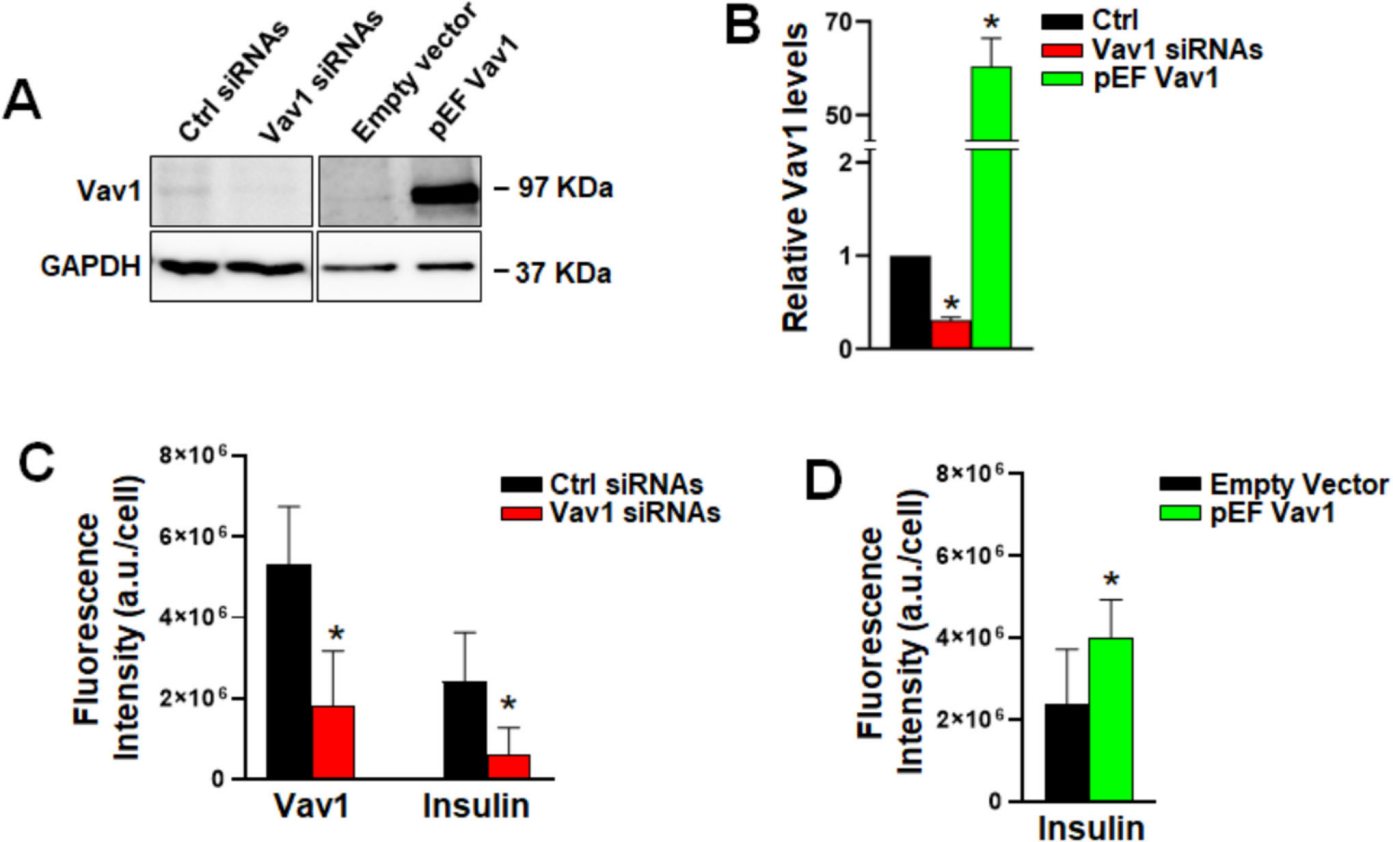
Effects of Vav1 modulation on insulin levels in EP cells. **A** Representative immunochemical analysis using a specific anti-Vav1 antibody in cells subjected to Vav1 silencing (Vav1 siRNAs) or overexpression (pEF Vav1). A non-silencing RNA (Ctrl siRNAs) and an empty vector (Empty vector) were used as controls. Relative amounts of Vav1 with respect to control, as determined by densitometric analysis of immunochemical bands, are shown in **B**. **C** Fluorescence intensity of Vav1 and insulin obtained from the simultaneous immunocytochemical analysis of the two proteins in EP cells in which Vav1 was silenced. **D** Fluorescence intensity of insulin in EP cells in which Vav1 was overexpressed. Data represents the mean of three independent experiments ± SD. *p < 0.05 compared to specific controls

After transient transfection with Vav1 siRNAs or with a construct expressing human Vav1, cells were subjected to Western blot analysis to assess the effectiveness of both modulation strategies (Fig. 4A, B). Transfected cells were then subjected to simultaneous immunocytochemical analysis of Vav1 and insulin to measure the expression of both proteins in the same cells. Fluorescence intensity analysis performed after 48 h post-transfection showed a significant reduction of Vav1 fluorescence in siRNA-transfected cells compared to control differentiating cells, confirming that the increase observed under control conditions had been blocked. This was accompanied by a considerable decrease in insulin staining within the same cell population, (Fig. 4C). These results indicated that the silencing of Vav1 to counteract its physiological increase negatively impacts insulin production, suggesting that Vav1 supports cell events leading to the progression of EP cells to β cells.

When EP cells were forced to overexpress Vav1, a strong increase of this protein was observed (Fig. 4A, B), making it difficult to simultaneously evaluate Vav1 and insulin in the same cells by immunocytochemical analysis. Therefore, cells overexpressing Vav1 were stained only with the anti-insulin antibody, revealing that, despite high variability within the cell population, a slight but statistically significant increase in insulin fluorescence was induced 48 h post-transfection (Fig. 4D). These findings indicate that maintaining elevated Vav1 levels for a few days during the differentiation of endocrine precursors into β cells is sufficient to induce a modest but significant enhancement in insulin production, supporting the hypothesis that Vav1 plays a role during a specific phase of the differentiation process from pancreatic precursors to insulin-producing cells.

### Effects of Vav1 modulation on PDX1 and miR-375 levels

**Fig. 5:**
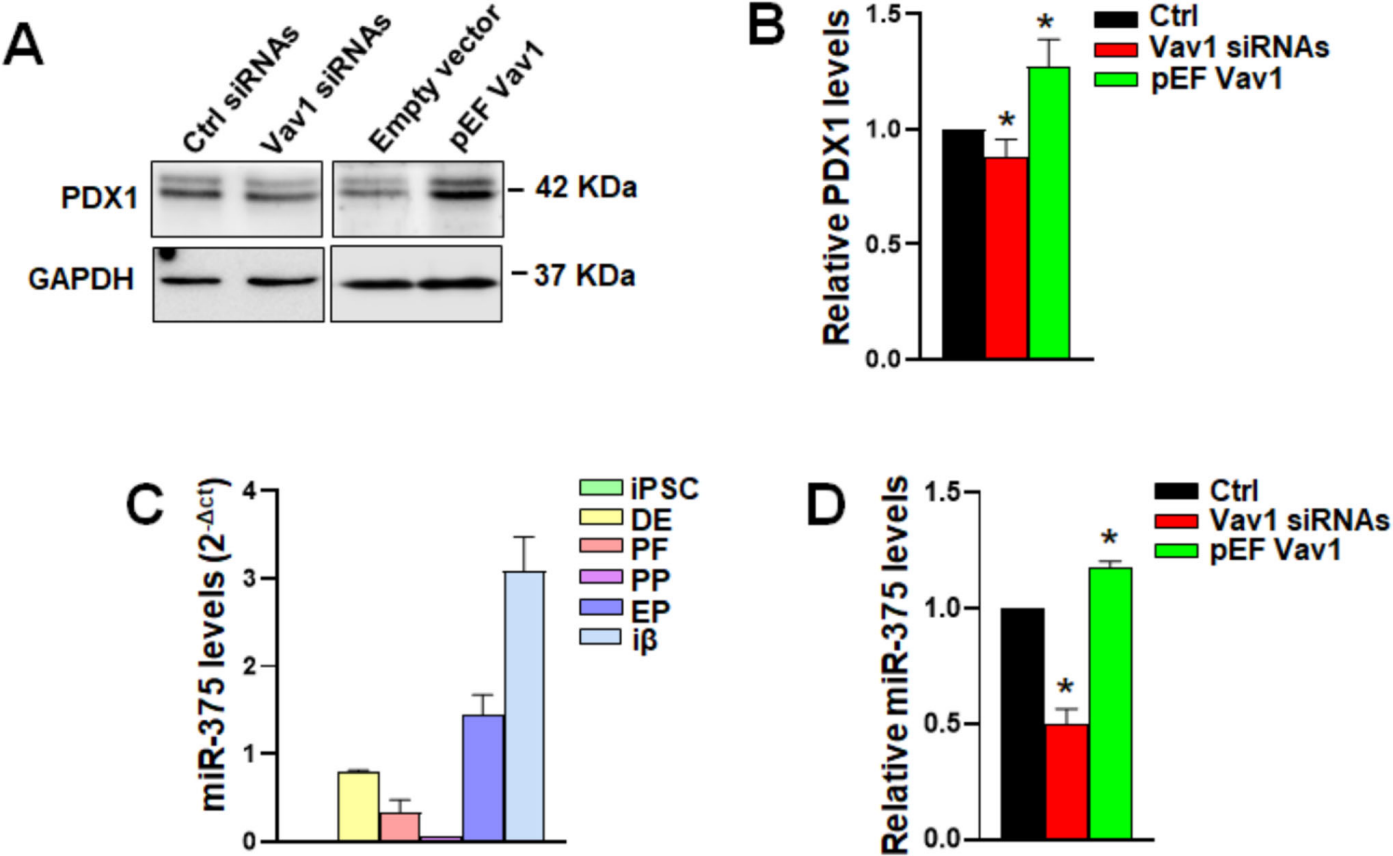
Effects of Vav1 modulation on PDX1 and miR-375. **A** Representative immunochemical analysis using a specific anti-PDX1 antibody in EP cells subjected to Vav1 silencing (Vav1 siRNAs) and overexpression (pEF Vav1). **B** Protein levels as determined by densitometric analysis of Western blot bands normalized to GAPDH. **C** miR-375 levels at distinct stages of iPSC differentiation into β cells, obtained using the 2^−ΔCt^ method. **D** RT-qPCR analysis of miR-375 levels in EP cells subjected to Vav1 silencing and overexpression, obtained using the 2^−ΔΔCt^ method. Data are the mean of three independent experiments ± SD. *p < 0.05 compared to specific controls (Ctrl) taken as 1

In the trans-differentiation of pancreatic adenocarcinoma derived HPAF2 cells to insulin producing cells, we previously demonstrated that Vav1 sustains the ATRA-induced expression of PDX1 [35], a key regulator of insulin gene transcription [37], which decreased in the latest stages of our differentiation process (Fig. 1B). Stemming from this observation, we evaluated whether Vav1 plays a similar role during the generation of insulin-producing cells from hiPSCs. We found that silencing Vav1 before its peak (day 16) at the EP stage induced a slight but significant reduction in PDX1 levels, while overexpression of Vav1 immediately after its peak (day 19) induced a significant increase in its expression compared to control differentiating cells (Fig. 5A, B).

Having established the relevance of Vav1 for insulin and PDX1 regulation in this differentiation procedure and based on our previous findings that Vav1 is involved in the regulation of miRNAs in other experimental models [24–26, 29], we investigated the possible role of Vav1 in modulating the levels of miR-375, a well-known regulator of pancreatic β cells fate by acting at various levels. Specifically, mir-375 is involved in both the production and secretion of insulin [33] and its expression is dependent from PDX1, which is in turn down-regulated by the miRNA [34, 42]. RT-qPCR analysis revealed dynamic miR-375 expression in our differentiation model (Fig. 5C) confirming literature data on ESCs differentiation [34]. In endocrine progenitors where the protein was forcibly modulated, silencing Vav1 induced a substantial decrease in miR-375 levels compared to control differentiating cells, while overexpression led to a slight but significant increase in miRNA expression (Fig. 5D). These findings suggest that during the differentiation of pancreatic precursors into insulin-producing cells, Vav1 modulates PDX1 and miR-375 levels, which are involved in regulating insulin production.

### Effect of Vav1 forced modulation on Akt serine/threonine kinase

**Fig. 6:**
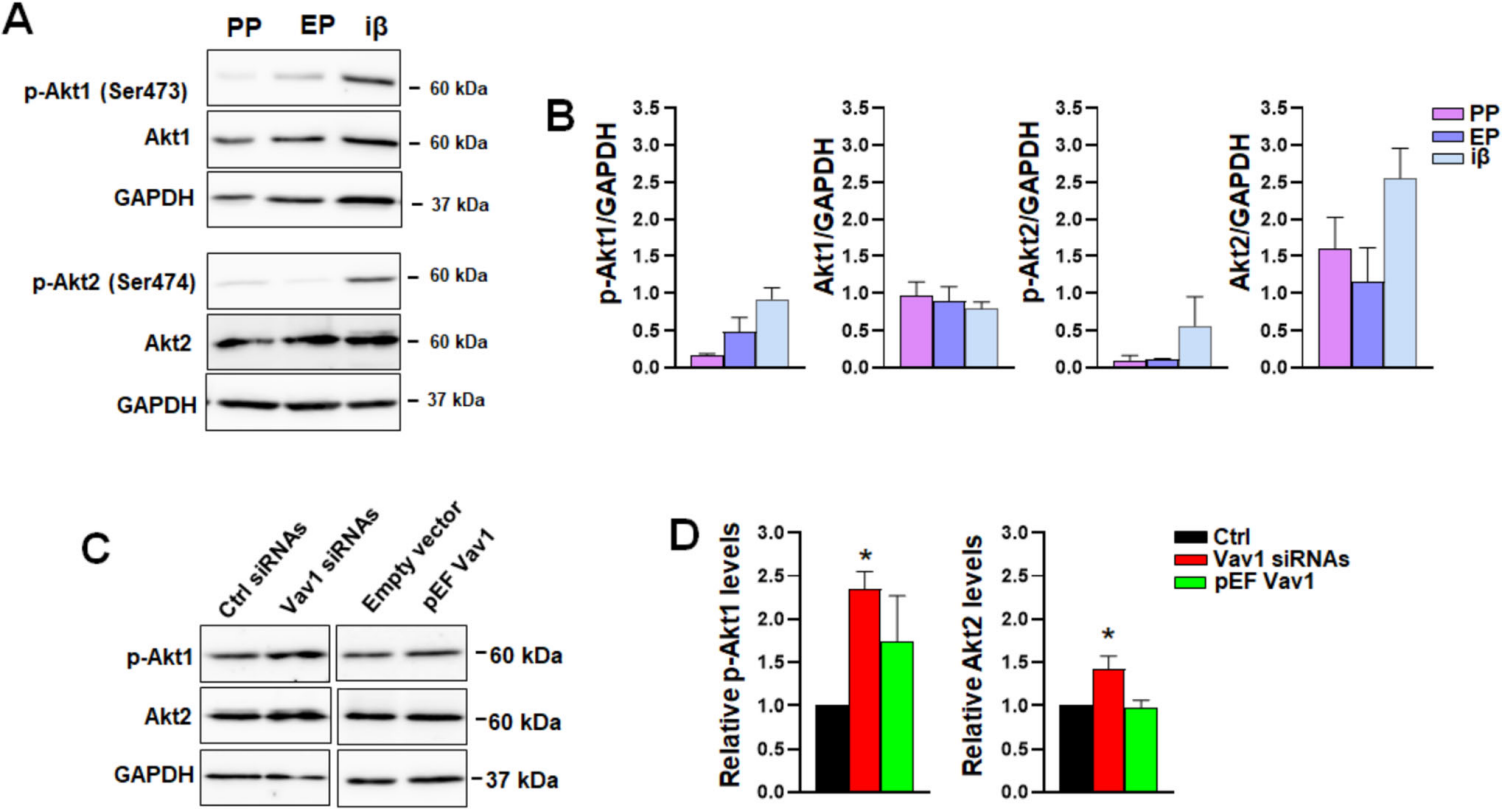
Effects of Vav1 modulation on Akt. **A** Representative immunochemical analysis using the indicated antibodies in differentiating cells at the PP, EP and iβ stages. **B** Protein levels as determined by densitometric analysis of Western blot bands normalized to GAPDH. **C** Representative immunochemical analysis of the indicated proteins in EP cells subjected to Vav1 silencing (siRNA) and overexpression (pEF Vav1). Protein levels, as determined by densitometric analysis of Western blot bands normalized to GAPDH, are shown in **D**. Data represents the mean of three independent experiments ± SD. *p < 0.05 compared to specific control, taken as 1

Considering that Vav1 can control the expression and/or activation of specific Akt isoforms in various cell models [27–29], we assessed whether the Akt family, also involved in β cells mass and in PDX1 stability [31, 43, 44], can be modulated by Vav1 during the differentiation of PP into insulin-producing cells. We analyzed the expression and activation status of the Akt1 and Akt2 isoforms, known to be modulated by Vav1 and described to play crucial roles in β cells [45]. Western blot analysis revealed a progressive increase in Ser-473 phosphorylation of Akt1 from PP to iβ cells, while an increase in Ser-474 phosphorylation of Akt2 was observed in cells reaching the β-like stage (Fig. 6A, B). Furthermore, no significant modification in the Akt1 levels was revealed, while Akt2 expression significantly increased in the final differentiation stage (iβ) (Fig. 6A, B).

Regarding the possible role of Vav1 in modulating Akt status, Vav1 overexpression did not significantly affect p-Akt1 levels, whereas silencing of Vav1 resulted in a notable increase in phosphorylated Akt1. Concerning the expression of Akt isozymes, silencing Vav1 induced a significant upmodulation of Akt2, while Akt1 levels remained unchanged (Fig. 6C, D).

## Discussion

The development of efficient therapies is essential to address the growing global number of individuals affected by T1D, and the generation of implantable insulin-producing cells from various precursors represents a promising opportunity to endogenously restore insulin production. In this context, established protocols that mimic endocrine pancreas development have enabled researchers to obtain high numbers of insulin-producing cells from human induced pluripotent stem cells *in vitro* [12–14]. However, the persistence of cells that retain stem properties can pose problems after transplantation [15]. This issue stems from the complex machinery driving the maturation of precursors from various origin to β cells, which is not yet fully understood at the genomic or protein levels.

Given the need for a better knowledge of the intracellular mechanisms involved in the maturation process to obtain fully functional β cells from induced pluripotent precursors, the present study explored the potential role of Vav1, a multidomain protein historically attributed to hematopoietic cells or solid tumors [16, 20], but also expressed by some β cells in pancreatic islets [35]. Our previous data revealed that Vav1 participates in initial stages of *in vitro* differentiation of multipotent precursors from biliary tree into β cells and in the partial trans-differentiation of PDAC-derived cells into insulin-producing cells [35]. Using a well-described 7-step differentiation protocol [40], we demonstrated here that the relatively high expression of Vav1 in iPSCs decreased during their differentiation up to the pancreatic precursors stage. Subsequently, Vav1 expression increases in cells driven towards the endocrine lineage, followed by a slight decrease in β-like cells. This suggests that Vav1 is not involved in the process driving human iPSCs to early pancreatic progenitors, but plays a role in pancreatic precursor cells that have adopted the endocrine lineage.

Despite the high heterogeneity of cell populations, confocal analysis allowed us to assess the presence of Vav1 inside the nuclear compartment and establish that the increase of Vav1 that characterizes cells at the endocrine progenitor stage mainly concerns its cytoplasmic portion. On the other hand, both nuclear and cytoplasmic Vav1 levels seem to decrease during the transition from endocrine progenitors to β-like cells. This suggests a stage-related role for Vav1 in cytoplasmic and/or nuclear compartments, consistent with the various functions of this multidomain protein described in different cell models [46].

To assess the possible role of Vav1 in our differentiation procedure, we modulated the protein to counteract the physiological changes occurring during the transition from pancreatic precursors to insulin-producing cells. We demonstrated the requirement of adequate levels of Vav1 for the generation of insulin-producing cells, confirming, in this differentiation model, our previous results obtained with biliary tree precursors and PDAC-derived cells [35]. This result apparently makes it difficult to correlate the levels of Vav1 and insulin measured during the differentiation process, as insulin production was detected in a maximum of 35% of cells at the iβ stage. On the other hand, in line with our previous findings [40], although only a fraction of cells expressed insulin, most of the remaining population at the end of the differentiation process was composed of endodermal or pancreatic progenitor cells with negligible expression of pluripotency markers, confirming the lineage restricted differentiation trajectory and the biological relevance of our data. Reflecting incomplete maturation in part of the culture, with cells that reach various levels of differentiation into insulin-producing cells, our results highlight the need of Vav1 only in specific stages of the maturation process and identify this protein as a potential factor to be modulated to optimize the differentiation procedure.

In addition to insulin, we revealed that Vav1 sustains the expression of PDX1, a transcription factor playing a pivotal role in β cell development as a primary regulator of insulin gene expression [36, 37]. This result correlates with the decrease of both proteins observed in the final stage of differentiation, where only a portion of cells reach full maturation. Considering that in PDAC-derived cells, Vav1 is essential for the expression of PDX1 induced by all-*trans*-retinoic acid (ATRA) [35], this result suggests that Vav1 could sustain ATRA activity during specific stages of the generation of insulin producing cells from hiPSCs, as ATRA is present in the culture medium at precise steps of the differentiation procedure [40].

Moreover, in endocrine progenitor cells in which Vav1 was silenced or overexpressed, we observed the down- or up-modulation of miR-375, a PDX1 target crucial for determining β-cell mass and insulin production and secretion during pancreatic differentiation [32–34, 42]. Although we were unable to selectively modulate cytoplasmic and nuclear Vav1, the presence of the protein inside the nuclear compartment suggests its possible involvement in gene transcription regulation and/or mRNA processing, as demonstrated in leukemia and breast tumor-derived cells [23–25, 28, 47]. While we could not establish the exact sequence of events, as the expression of miR-375 can be positively regulated by PDX1, in turn down-regulated by the miRNA, our data clearly indicate that Vav1 is involved in the complex machinery regulating expression of insulin, impacting two crucial molecules in generating fully functioning β cells [33, 36]. This suggests that modulating Vav1 levels could enhance the differentiation of insulin-producing cells generated from hiPSCs and offers a promising hint for strategies for producing β cells *in vitro*.

**Fig. 7:**
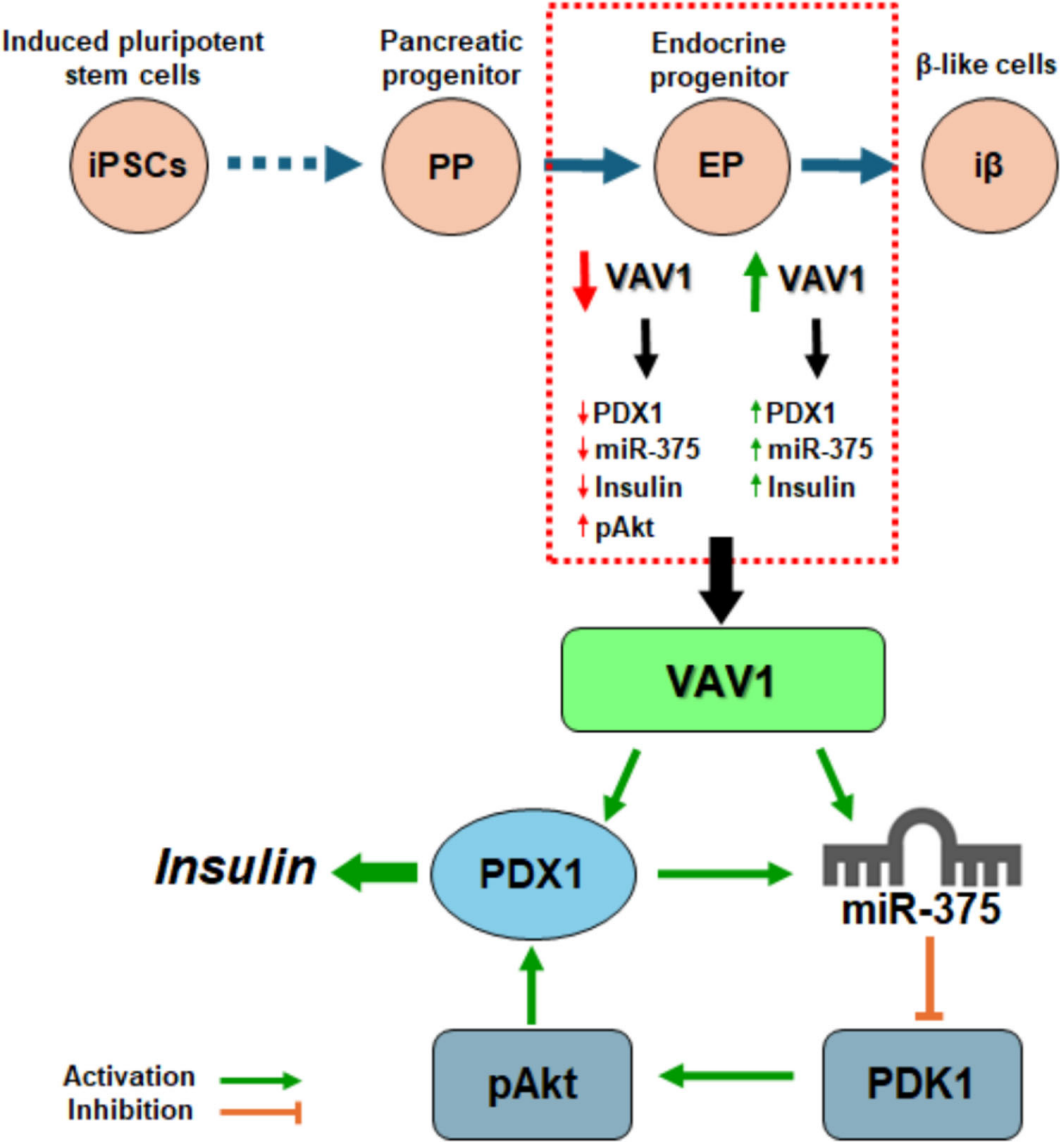
Schematic representation of the molecular pathway potentially regulated by Vav1 during the transition from pancreatic endocrine progenitors to insulin-producing cells

To better understand the intracellular impact of Vav1 modulation in our *in vitro* procedure to obtain insulin-producing cells, we considered that in mature β cells, miR-375 directly targets Pdk1, resulting in down-modulation of Akt activation, which regulates survival, proliferation, apoptosis and insulin secretion in β cells and constitutes a promising target in diabetes [48]. As Akt sustains the stability of PDX1 through a mechanism involving GSK3β [44], miR-375 could indirectly down-regulate PDX1 via Akt signaling. Of the three Akt isoforms, both Akt1 and Akt2 are expressed in β cells, with partially redundant functions, being both responsible of maintaining the islet mass [45, 48, 49]*.* On the other hand, proliferation, and apoptosis of β cells are significantly stimulated and inhibited, respectively, by Akt1, while defects in Akt2 levels mainly cause decreased insulin secretion [45, 49]. Furthermore, activation and/or expression of Akt1 and Akt2 are downregulated by Vav1 in various cell models [27–29], supporting the search for a Vav1/Akt relationship in our model of β cells generation. We revealed that an increase in activated Akt1 accompanies the transition of pancreatic precursor to β-like cells, while only the final maturation stage is characterized by increased expression and activation of Akt2. Both physiological and forced reduction of Vav1 in differentiating cells correlated with increased Akt activation, potentially due to the concomitant decrease in miR-375, suggesting the existence of a Vav1/PDX1/miR-375/Akt axis, summarized in Fig. 7, involved in the complex network producing fully functional β cells. On the other hand, the increased expression of Akt2 reflects the ability of Vav1 to down-modulate this Akt isoform in both breast cancer and PDAC derived cells, and the inverse relationship between Akt2 levels and nuclear Vav1 presence in the same cell models [28, 29].

Future research should focus on further elucidating the molecular mechanisms underlying the role of Vav1 in β cell differentiation. Investigating the interactions between Vav1 and other signaling pathways involved in β cell development could provide deeper insights into the regulatory networks governing generation of insulin-producing cells.

From a translational perspective, our findings suggest that targeting the Vav1/PDX1/miR-375/Akt axis could represent an innovative strategy to improve the efficiency and reproducibility of β cell differentiation from hiPSCs. Modulating Vav1 expression at specific stages may promote a more synchronized maturation of endocrine progenitors, enhancing insulin expression and reducing the persistence of undifferentiated cells. In this context, the combination of Vav1-targeted modulation with optimized differentiation protocols could further increase the yield of functional insulin-producing cells suitable for transplantation, as well as improving long-term viability and function of implanted hiPSC-derived β cells.

Moreover, since insulin protects pancreas islets from apoptosis via PDX1 [50], Vav1, regulating both molecules, could be of particular interest in pre-diabetic stages, where it could function as a marker and/or a target to support the expression of crucial molecules in insulin production. Additionally, exploring the potential of Vav1 modulation in other stem cell models and its effects on long-term β cell function and survival post-transplantation would be valuable.

In conclusion, the findings of this study suggest that strategies aimed at modulating Vav1 levels could enhance the differentiation of human iPSCs into functional β cells, offering a promising starting point for improving the generation of insulin-producing cells *in vitro*. This may have significant implications for regenerative medicine and diabetes therapy, potentially leading to more effective and sustainable treatments that reduce reliance on exogenous insulin and improve the quality of life for individuals with T1D.

## Data Availability

All data supporting the findings of this study are available on paper.
